# Increased expression of NCAPG (Non-SMC condensing I complex subunit G) is associated with progression and poor prognosis of lung adenocarcinoma

**DOI:** 10.1080/21655979.2022.2035124

**Published:** 2022-03-07

**Authors:** Xiaodong Wang, Xia Tian, Xufang Sui, Xiangfeng Li, Xiaoyang Zhao, Kai Han, Lingyan Sun, Yujin Dong

**Affiliations:** a Department of Oncology, Zibo City Fourth People’s Hospital, Zibo, Shandong, China; b Department of Breast and Thyroid Surgery, Zibo Central Hospital, Zibo, China; c Department of Radiology, Zibo City Fourth People’s Hospital, Zibo, Shandong, China; d Department of Surgery, Zibo City Fourth People’s Hospital, Zibo, Shandong, China; e Department of Internal Medicine, Zibo City Fourth People’s Hospital, Zibo, Shandong, China; f Department of Oncology, Zibo Central Hospital, Zibo, China

**Keywords:** NCAPG, LUAD, biomarker co-expression, cell cycle

## Abstract

Recently, studies have shown that the up-regulation of Non-SMC Condensin I Complex Subunit G (NCAPG) in some tumors can promote tumor progression, and its high expression has a strong correlation with the poor prognosis of patients. However, there are few studies on NCAPG in lung adenocarcinoma (LUAD). Our research is to explore the role of NCAPG in LUAD and try to reveal the possible molecular mechanism. We use public databases and tissue samples from LUAD patients to verify that NCAPG is significantly up-regulated in LUAD, and the high expression of NCAPG is related to the poor prognosis of patients. Subsequently, we found that silencing NCAPG can inhibit the proliferation and invasion of LUAD cells in vitro and the growth of subcutaneous tumors in nude mice in vivo. In order to explore the possible molecular mechanism of NCAPG’s function, we found out the genes co-expressed with NCAPG through the cBioportal database, and discovered that these genes were significantly enriched in the cell cycle and other pathways through DAVID analysis, which implies the importance of NCAPG in the cell cycle. Finally, we confirmed by flow cytometry that NCAPG affects the conversion of cell cycle mitosis from G1 to S. Taken together, our research results suggest that NCAPG plays a role in the progress of LUAD. Moreover, NCAPG can be used as a potential biomarker for the diagnosis of LUAD, as well as a potential therapeutic target for patients with LUAD.

## Graphical abstract

**Figure:**
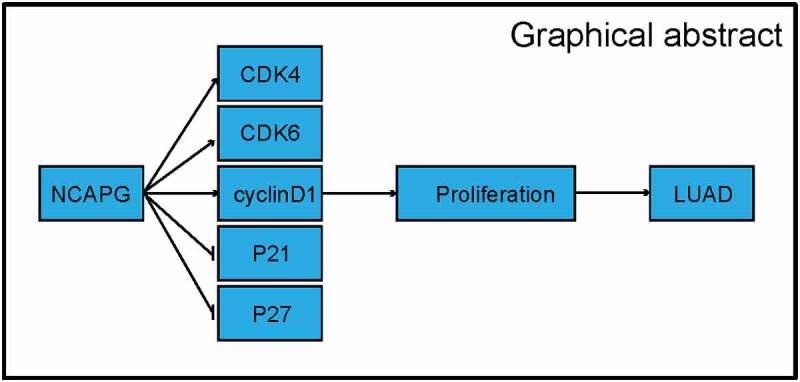

## Introduction

Although the level of diagnosis and treatment has been improving in recent years, lung cancer is still one of the malignant tumors with the highest morbidity and mortality in the world. The most common type of lung cancer cases is non-small cell lung cancer (NSCLC), which accounts for 80–85% of the total number of lung cancers. Specifically, NSCLC mainly includes adenocarcinoma, squamous cell carcinoma and large cell carcinoma. Among these types, LUAD has the highest incidence. Although more and more tumor biomarkers are being discovered, the current diagnosis of early stage of LUAD still lacks specific symptoms and biomarkers. Like other tumors, many patients with LUAD are already at an advanced stage when they are diagnosed, and are accompanied by lymph node and parenchymal organ metastasis, such as bone metastasis, brain metastasis, and liver metastasis. This makes many patients miss the best time for surgical treatment. Therefore, exploring new specific biomarkers and illustrate their possible mechanisms of LUAD is particularly important for precise and individualized treatment of patients with LUAD.

Non-SMC condensin I complex subunit G (NCAPG)is a mitotic associated chromosomal condensing protein, which is responsible for the condensation and stabilization of chromosomes during mitosis and meiosis. Phosphorylation of NCAPG activates the condensin complex [1]. There are pseudogenes for NCAPG gene on chromosomes 8 and 15.

Studies have shown that the abnormally high expression of NCAPG was closely related to the occurrence and development of many tumors and drug resistance [2,3]. Moreover, the overexpression of NCAPG is significantly positively correlated with the poor prognosis of patients [2,4]. Wu et al clarified that as an oncogenic factor, NCAPG was significantly up-regulated in hepatocellular carcinoma. At the same time, the up-regulated NCAPG promoted tumor cell proliferation and inhibited apoptosis by activating the PI3K/AKT/FOXO4 signal axis [5].

As we all know, cyclin not only plays a vital role in the normal physiological activities of cells, but its abnormal expression is also an important cause of tumor occurrence and progression. Studies have shown that NCAPG can also regulate the cell cycle by regulating the overexpression of cyclins including CDK4, CDK6, Cyclin D1, and the down-regulation of cell cycle inhibitors including P21 and P27, thereby inducing cardia Adenocarcinoma tumorigenesis [6]. Epithelial-mesenchymal transition (EMT) also plays a key role in tumor cell migration/invasion and various cancer processes, and is a vital step in tumor cell metastasis. Jia et al pointed out in their study that NCAPG promoted EMT through the Wnt/β-catenin signaling pathway to accelerate the metastasis of cardia cancer cells [7].

Throughout the history of tumor treatment, drug resistance has become the most influential factor in the treatment effect. After the introduction of chemotherapy drugs, drug resistance has plagued doctors and patients for more than half a century. In the current era of molecularly targeted drugs and personalized medicine, drug resistance is still a difficult problem to deal with. The mechanism of tumor resistance growth is also complex and diverse [8–12]. Song et al clarified that the significantly up-regulated NCAPG confered trastuzumab resistance to HER2-positive breast cancer by activating the SRC/STAT3 signal axis, which illustrated that NCAPG also played a non-negligible role in tumor resistance. Targeting NCAPG therapy may restore the sensitivity of tumor therapy to a certain extent [13].

Nevertheless, the role and clinical application value of NCAPG in lung cancer, especially LUAD, have not been elucidated. In our study, we found that NCAPG was abnormally highly expressed in LUAD. Overexpression of NCAPG often implies a poor prognosis for patients. Through in vivo and in vitro experiments, we further proved that knocking down NCAPG can inhibit tumor cell proliferation, migration and invasion, and inhibit the growth of subcutaneous xenograft tumors in nude mice. In clinical applications, NCAPG can be used as an effective biomarker for LUAD, and may serve as a potential target for the treatment of patients with LUAD.

This study aimed at evaluating the expression levels of NCAPG in LUAD tissues, and to initially explore the role of NCAPG in LUAD, and hopes to provide a certain degree of theoretical guidance for the diagnosis and treatment of LUAD.

## Materials and methods

### Tissue specimens

In our study, a total of 60 LUAD patients and 30 adjacent tissues were collected. Each patient did not receive radiotherapy or chemotherapy before surgical resection. And patients involved in our research signed an informed consent form before receiving surgery. These tissue samples were taken and immediately immersed in formalin solution. It was then stored in a liquid nitrogen tank at −80°C. The research was approved by the Ethics Committee of Zibo Central Hospital and followed the ethical standards of the responsible committee on human experimentation (institutional and national) and with the Helsinki Declaration of 1975.

## Cell culture and transfection

All cell lines used in our study were purchased from Shanghai institute of life sciences. These cell lines were cultured in a 5% CO_2_ cell incubator at 37°C with DMEM medium (Gibco) containing 10% fetal bovine serum (Biological Industries). NCAPG knockdown were performed using two small interfering RNA (siRNA) targeting NCAPG mRNA according to the operation manual. siNCAPG#1 sense: 5′-GGAGUUCAUUCAUUACCUUTT-3′ and antisense: 5′-AAGGUAAUGAAUGAACUCCTT-3′; siNCAPG#2 sense: 5′-GCUGAAACAUUGCAGAAAUTT-3′ and antisense: 5′-AUUUCUGCAAUGUUUCAGCTT-3′

## Immunohistochemistry

Immunohistochemical (IHC) experiment was used to detect the expression level of NCAPG in LUAD patients and adjacent tissues. At the same time, we explored the expression of NCAPG and Ki-67 in nude mice subcutaneously transplanted tumor samples. IHC results are determined according to the degree of staining of cells and the proportion of positive cells in the section: cell staining tan is recorded as 3 points, brown as 2 points, yellow as 1 point, and no staining as 0 points. In addition, randomly observe 5 high magnification fields (200X), and count 100 cells in each field. Count the proportion of the number of stained positive cells in 500 cells. <5%, count as 0 points; 6%-25%, count as 1 point; 26%-50%, count as 2 points; 51%-75%, count as 3 points; >75%, count as 4 points. The two scoring results are added together, the final score is 0 and it is regarded as negative, 1–2 points as weak positive, 3–5 points as positive, and >5 points as strong positive. Antibody involved including NCAPG (ab251864, 1:50 dilution) and Ki-67 (ab92742, 1:500 dilution). Specific experimental steps were performed followed the manufacturer’s instructions.

## RNA isolation, reverse transcription and Real-Time PCR

Total RNA was extracted using TRIzol (Solarbio) reagent after the cells filled the culture flask. After measuring the RNA concentration, 5ug RNA was reversed into cDNA using a reverse transcription kit (Thermo) according to the manufacturer′s instructions. Subsequently, the products were subjected to quantitative Real-Time PCR using FastStart Universal SYBR Green Master (Roche, Sigma-Aldrich). GAPDH was used as an internal control. The primers used in our research were listed as follow: GAPDH: forward 5′-CTGGGCTACACTGAGCACC-3′, reverse 5′-AAGTGGTCGTTGAGGGCAATG-3′; NCAPG forward 5′-ATCCAGAAGTTAGACGGGCAG −3′, reverse 5′-GTGCGCCCTACAATTTTTGGC-3′.

## Protein extraction and Western blot

Use RIPA to lyse cells to obtain total protein. After measuring the protein concentration, follow-up experiments were carried out according to the quality of 30ug per well. The total protein was first separated with a 10% SDS-PAGE gel (Solarbio), and then electroporated onto the PVDF membrane. After the electroporation, the membrane was blocked with 5% skim milk for 1 hour at room temperature. Then the PVDF membrane was incubated with the specific primary antibody at 4°C overnight. Incubate the membrane with a secondary antibody conjugated with horseradish peroxidase for 1 hour at room temperature on the second day. Finally, the blots were detected with enhanced chemiluminescence (Vector Laboratories, Burlingame, CA). Antibodies used for Western blot included GAPDH (ab9485, 1:2500), CDK4 (ab108357, 1:5000), CDK6 (ab124821, 1:50,000), cyclinD1 (ab16663, 1:100), P21 (ab109520, 1:5000), P27 (ab2034, 1:5000) and NCAPG (ab155553, 1:2000).

## MTT, CCK8, BrdU assay

For 3-(4, 5-Dimethylthiazol-2-yl)-2, 5-diphenyltetrazolium bromide (MTT) assay, the cells were plated into a 96-well plate at a density of 6 × 10^3^ per well, with 6 replicates per group. Regarding cells adherence to the wall as day zero, the absorbance of cells at 490 nm were detected on the zero day, the first day, the second day, the third day respectively to evaluate the cell proliferation ability. For CCK8 assay, the cells were seeded in a 96-well plate at a density of 1 × 10^3^ per well. Similarly, fresh medium containing 10ul CCK8 reagent was added to each well. After incubated at 37°C for 1 hour in incubator containing 5% CO_2_. Measure the absorbance at 450 nm at the time point of the day zero, first, second and third respectively.

For BrdU assay, 1 × 10^3^ cells per well were plated into the 96 well plate with 6 replicates per group. Wash the adherent cells three times with PBS. Then detect the proliferation ability of each group cells using the BrdU cell proliferation kit (Cell Signaling Technology) following the manufacturer’s instructions. Finally, the absorbance value was measure at the wavelength of 450 nm.

## Colony formation assay

LUAD cells of different treatment groups were seeded in six-well plates at a density of 1 × 10^3^ per well. After the cells were cultured in the incubator for two weeks, the medium was discarded, and then the cells were fixed with 4% paraformaldehyde (Biosharp) at 4°C. Finally, the fixed cells were stained with crystal violet at room temperature. Image J software was used to count the number of clones.

## Transwell assay

First, the cells were starved in serum-free DMEM medium for 6 hours. Then plated the cells into the upper chamber of the transwell chambers (Corning) with 200 μl serum-free medium, and the lower chamber contained a complete medium containing 20% fetal bovine serum. After cultured at 37°C with 5% CO2 in incubator for 3 days, cells were fixed with 4% paraformaldehyde for 30 minutes at 4°C, followed by staining with crystal violet for 30 minutes at room temperature. Finally, use Image J to analyze the results.

## Immunofluorescence (IF)

In order to explore the location of NCAPG molecules in cells, immunofluorescence experiment was performed according to the manufacturer’s manual. The NCAPG antibody was purchased from abcam company (ab226805). The Fluorescein Isothiocyanate labeled donkey anti-rabbit antibody was used to mark NCAPG. The cell nucleus was stained with 4′,6-diamidino-2-phenylindole (DAPI). Capture the image with Olympus FV1000D microscope. Detailed method was performed as described [14].

## Flow cytometry

Take the lung adenocarcinoma cells in the growth stage, add 3 mL PBS to make a cell suspension, transfer to a 15 mL centrifuge tube, centrifuge at 1500rpm for 5 min, and remove the supernatant. Add 500 μL of PBS to gently pipette the cell cluster into a cell suspension, then add 2 mL of 70% cold ethanol, mix well and fix for 30 min. Add 5 mL PBS, centrifuge at 1500 rpm for 5 min, and remove the supernatant. Add 5 mL PBS to resuspend the cells, centrifuge at 1500rpm for 5 min, and remove the supernatant. Add 800 μL PI staining solution, gently blow the cell cluster with a gun, mix, and stain at room temperature for 30 minutes in the dark. Use flow cytometer to test cell cycle on the machine. Principle of the method was explained as described [15].

## In vivo experiment

The BALB/c-nude mice (5 weeks of age, 18–20 g) were purchased from Shanghai Experimental Animal Center, Chinese Academy of Sciences. In brief, 1 × 10^6^ A549 cells including the shCTRL group(N = 5) and shNCAPG group(N = 5) were injected subcutaneously into nude mice. The length and width of xenografts was measure and subjected to the formula V = (length*width^2^)/2 every other day, length refers to the longest diameter of the tumor volume, and width refers to the diameter of the widest part of the tumor. After the experiment finished, the BALB/c-nude mice were euthanized, and the xenografts were accurately weighed. Finally, the xenografts were embedded into paraffin for IHC experiment. All animal experiment procedures were approved by the Ethics Committee of Zibo Central Hospital.

## Co-expressed genes and enrichment analysis

The cBioPortal for Cancer Genomics (http://www.cbioportal.org) was applied to seek the co-expression genes of NCAPG through conducting a Spearman correlation analysis in Lung Adenocarcinoma database (TCGA, Firehose Legacy). We singled out the genes positively co-expressed with NCAPG (r ≥ 0.4, p < 0.01) for subsequent enrichment analysis in DAVID (https://david.ncifcrf.gov/).

## Statistical analysis

All data were presented as mean ± SD (standard deviation). One way or two ways ANOVA was used to calculated the comparisons between groups through GraphPad Prism 8 software. P values <0.05 were considered statistically significant. * P < 0.05, **P < 0.01, ***P < 0.001, ****P < 0.0001. Chi-square test was used to determine whether the patient’s clinical characteristics were related to NCAPG expression.

## Results

We aimed at evaluating the expression levels of NCAPG in LUAD tissues, and to initially explore the role of NCAPG in LUAD. In our research, we found NCAPG was up-regulated and related to the poor prognosis of LUAD. Silencing NCAPG could inhibit the proliferation and invasion of LUAD cells in vitro and the growth of subcutaneous tumors in nude mice in vivo. Finally, we confirmed by flow cytometry that NCAPG affects the conversion of cell cycle mitosis from G1 to S.

**NCAPG was over-expressed in LUAD tissues and cell lines and was associated with patients’ poor prognosis.**

**Figure 1.**
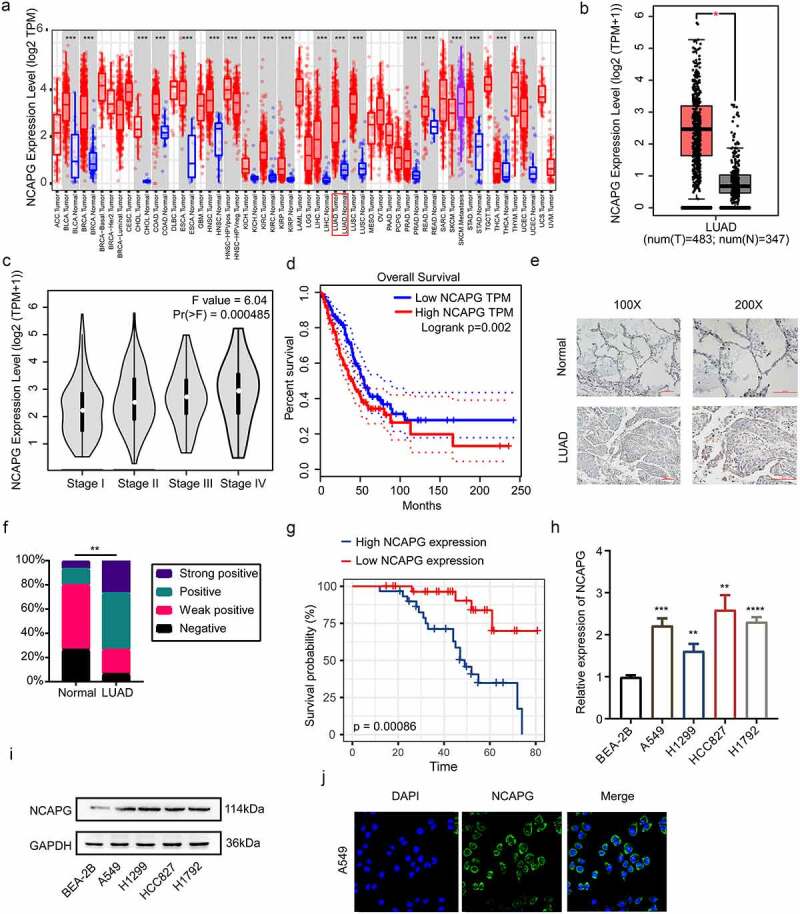
NCAPG was over-expressed in LUAD tissues and cell lines and was associated with patients’ poor prognosis. (a) the mRNA expression level of NCAPG in pan-cancer tissues and corresponding normal specimens by using the TIMER web tool. ***, p < 0.001. (b) the mRNA expression level of NCAPG in LUAD patient tissues (N = 483) and normal samples (N = 347) through GEPIA online tool. *, p < 0.05. (c) NCAPG mRNA levels at different stages of LUAD. (d) the overall survival time of LUAD patients with high or low expression level of NCAPG were analyzed by GEPIA. Logrank p = 0.002. (e) display image of IHC results of NCAPG in LUAD tissue (N = 60) and normal tissue (N = 30). (f) statistical analysis of IHC results in (E). **, p < 0.01. (g) analysis of survival probability of 60 cases of LUAD patients with different NCAPG expression level. p = 0.00086. (h-i) BEA-2B, A549, H1299, HCC827 and H1792 cells were harvested and subjected to Western blotting and RT-PCR analysis. Results presented as Mean ± SD (N = 3). **, p < 0.01, ***, p < 0.001, ****, p < 0.0001. (j) IF was performed to clarify the location of NCAPG in A549 cell. DAPI was used to label the nucleus.

Multiple studies have mentioned that NCAPG was highly expressed in a variety of solid tumors including liver cancer and breast cancer. However, the research on the relationship between NCAPG and NSCLC, especially LUAD, is still insufficient. Therefore, we first used the website tools TIMER(https://cistrome.shinyapps.io/timer/) and GEPIA(http://gepia.cancer-pku.cn/) based on TCGA data to explore the mRNA expression levels of NCAPG in tumor tissues and non-cancerous tissues [16,17]. The results indicated that compared to non-cancerous tissues, NCAPG was overexpressed in a variety of tumors including LUAD (Figure 1a,b). The results were statistically significant (p < 0.05). GEPIA was applied to further explore the relationship between the high expression of NCAPG and the clinical stage and overall survival of patients with LUAD. We found that as the disease progresses, the level of NCAPG gradually increases (Figure 1c). At the same time, patients with high NCAPG expression have a shorter overall survival time(p = 0.002) (Figure 1d). Furthermore, the tissue microarray containing 30 cases of non-tumor lung tissue samples and 60 cases of LUAD tissue samples were subjected to IHC experiment to assesses the expression level of NCAPG. Consistent with the mRNA expression of NCAPG in the TCGA database, NCAPG was significantly up-regulated at the protein level in LUAD according to the IHC (Figure 1e). The statistical results suggested that in normal tissues, the proportion of samples with positive and strong positive results of NCAPG staining was 80%, while the proportion of samples with LUAD was 73.33% (figure 1f). Subsequently, among the LUAD tissues, the negative and weak positive samples were regarded as the NCAPG low expression group, and the NCAPG staining positive and strong positive were defined as the NCAPG high expression group. After collecting the clinical information of these patients, we found that patients with high NCAPG expression have a worse prognosis(p = 0.00086) (Figure 1g). Moreover, the expression level of NCAPG in patients is related to clinical stage(p = 0.0392), T factor(p = 0.0000), lymph node metastasis(p = 0.008) and distance metastasis(p = 0.0135) (Table 1). Further cell experiments illustrated that compared with normal lung epithelial cells (BEA-2B), NCAPG was overexpressed in LUAD cell lines including A549, HCC827, H1299 and H1792(Figure 1h,i). To explore the location of NCAPG, IF experiment was performed in A549 cell line. Our results showed that NCAPG was mainly located in the cytoplasm, and a small part of it was located in the nucleus (Figure 1j).

**Table 1.** Relationship between NCAPG expression and clinical pathological characteristics of patients with lung adenocarcinoma

Clinicopathologic variables	NCAPG			*P*-value
	All cases (*N* = 60)	Low expression (*n* = 32)	High expression (*n* = 28)	
Age(years)				
X ≤ 60	36	20	16	0.6726
> 60	24	12	12	
Gender				
Male	48	25	23	0.6979
Female	12	7	5	
Clinical stage				
I+ II	21	15	6	0.0392
III+IV	39	17	22	
T factor				
T1+ T2	23	20	3	0.0000
T3+ T4	37	12	25	
Lymph node metastasis				
N0+ N1	31	23	8	0.008
N3+ N4	29	9	20	
Distance metastasis				
M0	47	29	18	0.0135
M1	13	3	10

## Silencing NCAPG inhibited the proliferation and invasion of LUAD cell lines in vitro

**Figure 2.**
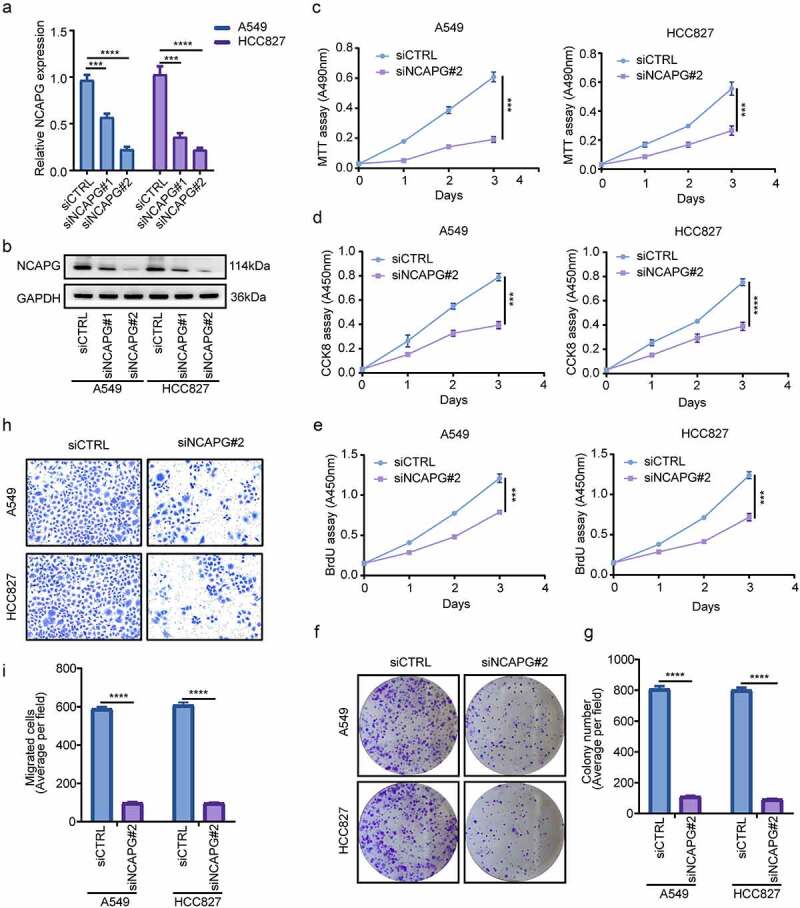
Silencing NCAPG inhibited the proliferation and invasion of A549 and HCC827 cell lines in vitro. (a-b) NCAPG was knocked down with small interfering RNA (siRNA) according to the manufacturer’s instructions. After 72 h, A549 and HCC827 cells were harvested and subjected to Western blot and RT-PCR to determine the efficiency of knockdown. ***, p < 0.001, ****, p < 0.0001. (c-f) MTT(c), CCK8(d), BrdU (e) and colony formation (f) assay were used to detected the proliferation ability after NCAPG was knocked down. ***, p < 0.001. (g) use Image J tool to count the number of clone-forming cells. ****, p < 0.0001. (h) Transwell assay was performed to determine the invasion of siCTRL group and siNCAPG#2 group cells. (i) Image J was applied to calculate the result of (H). ****, p < 0.0001. Data was shown in the form of mean ± SD (N = 3).

To further explore whether NCAPG has certain biological functions, we used small interfering RNA (siRNA) to inhibit the expression of NCAPG in the A549 and HCC827 cell lines, RT-PCR and Western blot were used to detect the knockdown efficiency at the mRNA and protein levels, respectively (Figure 2a,b). Subsequently, MTT, CCK8, BrdU cell proliferation experiments and colony formation experiments were performed to determine the effect of knocking down NCAPG on cell proliferation (Figure 2c-g). The results indicated that silencing NCAPG can inhibit cell proliferation. Furthermore, the outcomes of transwell experiments suggested that knocking down NCAPG suppressed the invasion of A549 and HCC827 cell line (Figure 2h,i).

## NCAPG knockdown suppressed the growth of subcutaneous transplanted tumors in nude mice

**Figure 3.**
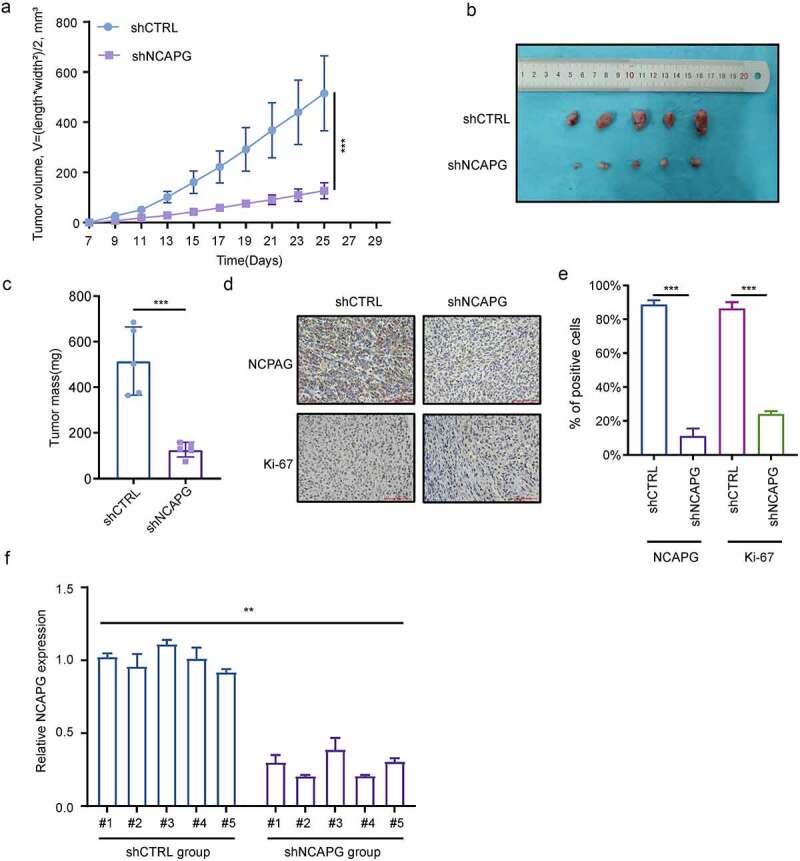
NCAPG knockdown suppressed the growth of subcutaneous transplanted tumors in nude mice. (a-c) A549 cells were transfected with short hairpin RNA (shRNA) targeting NCAPG (shNCAPG) and control shRNA (shCTRL). After 72 h, cells were injected subcutaneously into the nude mice for 25 days. The volume and mass of xenografts were measured in (a) and (c). The image of xenografts was shown in (b). ***, p < 0.001. (d-e) the NCAPG and Ki-67 staining through IHC were shown in (d) and (e). After the experiment finished, Total RNA of the xenografts was extracted for RT-PCR experiments. **, p < 0.01.

Subsequently, we constructed subcutaneous xenograft tumor nude mice model to evaluate the effect of NCAPG on tumor growth in vivo. We found that after knocking down NCAPG by shRNA, the tumor growth rate was significantly slowed down (Figure 3a). After the experiment finished, the mice were euthanized. We found that the tumor volume and mass of the nude mice in the shNCAPG group were smaller than those in the control group (Figure 3b,c). Meanwhile, IHC results showed that the number of Ki-67-positive cells in the tumors in the NCAPG knockdown group was markedly reduced (Figure 3d,e). We extracted RNA of the mice for RT-PCR experiments, and detected the NCAPG mRNA levels in the nude mice tumors of the shNCAPG group and the control group. The result was shown in the figure 3f. In summary, knocking down NCAPG could also inhibit tumor growth in vivo.

## Enrichment analysis of NCAPG co-expressed genes

**Figure 4.**
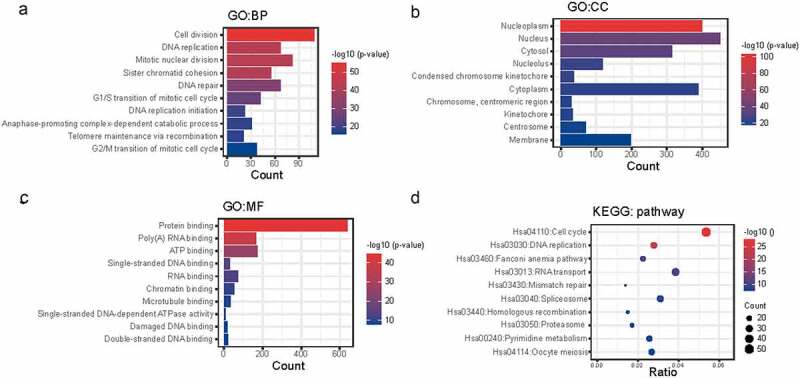
Enrichment analysis of NCAPG co-expressed genes. (a-d) the positively co-expressed genes of NCAPG (r ≥ 0.4, p < 0.01) gained through web tool cBioPortal were subjected to GO (a-c) and KEGG analysis (d). BP: Biological Process; CC: Cellular Component; MF: Molecular Function.

The co-expressed genes of NCAPG may have similar biological functions as NCAPG. To further clarify the role of NCAPG in LUAD, we utilized the website tool cBioPortal (http://www.cbioportal.org) to perform gene co-expression analysis. We brought those genes that were positively co-expressed with NCAPG (r ≥ 0.4, p < 0.01) into our study (Supplementary1). Then DAVID (https://david.ncifcrf.gov/)online website tool was used to perform functional enrichment analysis of these co-expressed genes (Supplementary 2–5). The results showed that cell division (GO:0051301), DNA replication (GO:0006260), mitotic nuclear division (GO:0007067), etc. were the top ten enriched biological processes (BP) (Figure 4a). Nucleoplasm (GO:0005654), nucleus (GO:0005634), cytosol (GO:0005829), etc. were the top ten enriched cellular components (CC) (Figure 4b). Protein binding (GO:0005515), poly(A) RNA binding (GO:0044822), ATP binding (GO:0005524), etc. were the top ten enriched molecular functions (MF) (Figure 4c). Cell cycle (hsa04110), DNA replication (hsa03030), fanconi anemia pathway (hsa03460), etc. were the top ten enriched Kyoto Encyclopedia of Genes and Genomes (KEGG) pathways (Figure 4d). These results indicated that NCAPG played a certain role in cell cycle and other pathways, which was consistent with our previous cell experiments.

## Knockdown of NCAPG arrested the G1 phase in LUAD cell lines

**Figure 5.**
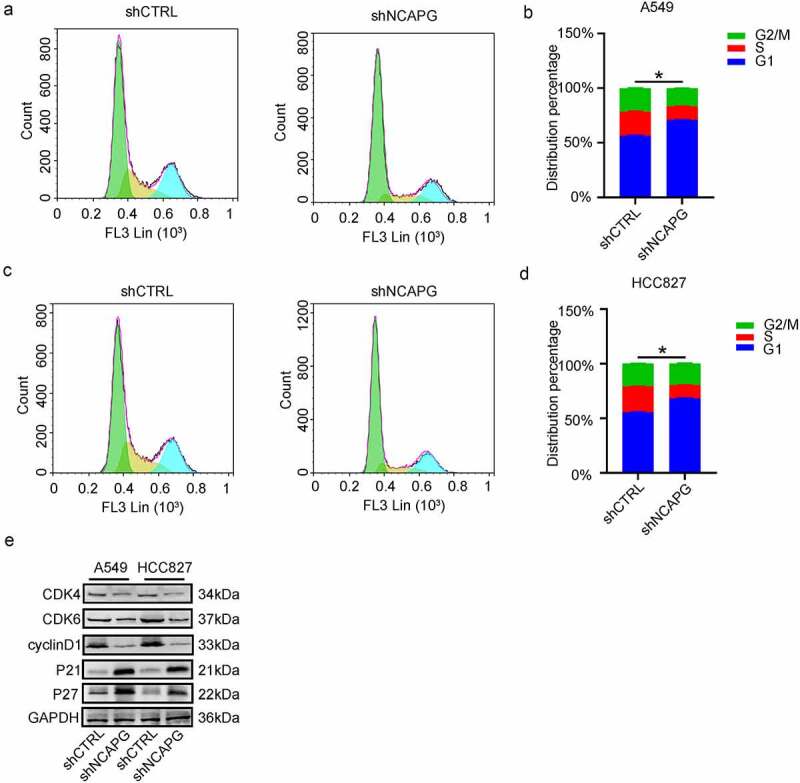
Knockdown of NCAPG arrested the G1 phase in LUAD cell lines. (a-d) The changes in each phase of the cell cycle after inhibiting NCAPG. (e) Changes in cell cycle-related proteins after NCAPG was knocked down. *, p < 0.05.

To further elucidate the potential mechanism of NCAPG affecting the proliferation of LUAD, considering that NCAPG co-expressed genes are significantly enriched in the cell cycle pathway, we performed flow cytometry to detect cell cycle changes after knocking down NCAPG. The results showed that inhibiting the expression of NCAPG can arrest the cell cycle of A549 (Figure 5a,b) and HCC827 (Figure 5c,d) cell lines in G1 phase. Then we can clearly see through the Western blot experiment that after knocking down NCAPG, the expression of CDK4, CDK6, and cyclinD1 were inhibited, while P21 and P27 were opposite, which explained the reason why the cell cycle was blocked in the G1 phase, and further expounded the specific molecular mechanism of NCAPG affecting cell proliferation (Figure 5e).

## Discussion

In order to explore the expression and potential function of NCAPG in lung cancer, especially in LUAD, and provide certain theoretical support for the diagnosis and treatment of LUAD in the future, our research results clarified that compared with normal lung tissue and lung epithelial cells, NCAPG mRNA and protein expression levels were significantly up-regulated in LUAD tissues and cells. Moreover, LUAD patients with high NCAPG expression have worse overall survival than patients with low NCAPG expression. Silencing NCAPG could markedly inhibit the proliferation, migration and invasion of LUAD cells in vitro, and inhibit tumor growth in vivo. To further investigate the potential molecular mechanism of NCAPG in LUAD, we used bioinformatics analysis to seek the positively co-expressed genes of NCAPG. Through functional and pathway enrichment analysis, we illustrated that the biological function of NCAPG was significantly correlated to cell cycle signaling pathway, which is consistent with our in vivo and in vitro experimental results. Finally, we confirmed by flow cytometry that NCAPG affects the conversion of cell cycle mitosis from G1 to S phase.

Due to the lack of specific symptoms and diagnostic biomarkers, many LUAD patients are already in a relatively advanced pathological stage when they are diagnosed, which makes the patients lose the best time for treatment. Therefore, looking for LUAD-specific biomarkers with diagnostic value is of great significance for early diagnosis of LUAD and providing suitable treatment options. Previous studies suggested that NCAPG was significantly up-regulated in a variety of malignant solid tumors including liver cancer, prostate cancer, gastric cancer, renal clear cell carcinoma, etc [2,18–21]. In the meantime, the elevated NCAPG was positively correlated with poor prognosis of these cancer patients. MicroRNAs (miRNAs) are endogenous non-coding RNAs with regulatory functions found in eukaryotes. Its role is mainly to recognize target mRNA through base complementary pairing, and guide the silencing complex to degrade target mRNA or inhibit the translation of target mRNA according to the degree of complementarity, and then participate in many processes of tumorigenesis and development [22,23]. Some researchers have also found that in some tumors, miRNA is also involved in regulating the expression of NCAPG. Arai T, et al indicated that miR-99a-3p can inhibit the progression of castration-resistant prostate cancer by regulating the mRNA level of NCAPG [24]. In hepatocellular carcinoma, Ai J suggested that microRNA-181c inhibited growth and metastasis by targeting NCAPG [25].

To sum up, NCAPG may become a potential molecular target for the treatment of some cancer patients including LUAD. By precisely targeting NCAPG, inhibiting NCAPG mRNA or protein levels may benefit some patients to a certain extent. As mentioned earlier, NCAPG can participate in tumor progression through PI3K/AKT, Wnt/β-catenin and other signaling pathways. At the same time, in HER2-positive breast cancer, NCAPG promotes the development of trastuzumab resistance by activating the SRC/STAT3 axis. This suggests that continuing to develop inhibitors targeting key protein molecules in these pathways may prolong the survival time of cancer patients with high expression of NCAPG, and restore the sensitivity of tumors to drug treatment.

In summary, our research reveals that NCAPG is significantly upregulated in LUAD. At the same time, silencing NCAPG can inhibit tumor progression. Compared with LUAD patients with low NCAPG expression, patients with high NCAPG expression have a poor prognosis. This suggests that NCAPG can be used as a biomarker for the diagnosis of LUAD. Meanwhile, NCAPG can serve as a potential molecular target for the clinical treatment of LUAD.

## Conclusion

This research indicated that NCAPG is upregulated in LUAD tissues and cell lines, and its high expression has a strong correlation with the poor prognosis of patients. Inhibition of NCAPG significantly restricted the proliferation and invasion of LUAD cell lines and tumor growth. Moreover, through bioinformatics analysis and experimental verification, we found that NCAPG can block the cell cycle in G1 phase and inhibit tumor proliferation and progression.

## Highlights

1. NCAPG is up-regulated and related to the poor prognosis of LUAD.
2. NCAPG can significantly promote the proliferation and invasion of LUAD cells.
3. Inhibition of NCAPG expression in LUAD cells arrested the G1 phase and thus inhibited cell proliferation.
